# The Association Between Health-Related Quality of Life and Bioelectrical Impedance-Derived Phase Angle in Community-Dwelling Older Adults: A Cross-Sectional Study

**DOI:** 10.7759/cureus.86054

**Published:** 2025-06-15

**Authors:** Tatsuhiko Asano, Masatsugu Okamura, Takuo Nomura

**Affiliations:** 1 Department of Rehabilitation Medicine, Kansai Medical University General Medical Center, Moriguchi, JPN; 2 Berlin Institute of Health Center for Regenerative Therapies (BCRT), Charité – Universitätsmedizin Berlin, Berlin, DEU; 3 Department of Rehabilitation Medicine, School of Medicine, Yokohama City University, Yokohama, JPN; 4 Faculty of Rehabilitation, Kansai Medical University, Hirakata, JPN

**Keywords:** bioelectrical impedance analysis, body composition, health-related quality of life (hrqol), muscle quality, physical functions, physical therapy, public health, rehabilitation

## Abstract

Background and objective

The phase angle (PhA) obtained using bioelectrical impedance analysis is a highly reliable indicator that reflects the quality of skeletal muscle, as it is calculated directly from reactance and resistance when an electric current is applied and does not use an estimation formula. Although PhA is a useful indicator of nutritional status and prognosis, its relationship with health-related quality of life (HRQoL) in older adults from the general population has not been fully investigated. In this study, we aimed to assess the relationship between HRQoL and PhA in community-dwelling older adults to determine whether PhA is a useful indicator of decreased HRQoL.

Methods

The study included 162 older adults (114 women; average age: 77.8 ± 5.5 years) who were independent in their daily activities and did not have serious musculoskeletal or internal disorders. The EuroQol 5-Dimension 5-Level (EQ-5D-5L) was used to evaluate HRQoL, and PhA was measured using a body composition analyzer (MC-780A-N; TANITA Corporation, Tokyo, Japan), with the left and right values averaged. In addition, we evaluated sex, BMI, appendicular skeletal muscle mass (ASM), skeletal muscle index, and physical function (grip strength, walking speed, knee extension strength, and five-times sit-to-stand test). We examined the relationship between PhA and each evaluation item using Spearman's rank correlation test, with HRQoL as the objective variable, using multiple regression analysis.

Results

The Spearman rank correlation test showed a significant correlation between HRQoL and PhA (ρ=0.2550), as well as with ASM, grip strength, and knee extension strength (ρ=0.1580, 0.1610, and 0.1670, respectively). In the multiple regression analysis, PhA was a significant independent factor explaining HRQoL (t=2.8097, p=0.0056); however, no association was found with the other evaluation items.

Conclusions

Based on our findings, PhA is an independent factor associated with HRQoL in community-dwelling older adults. Hence, PhA may be a useful indicator of the risk of decreased HRQoL. Compared with other physical function and muscle strength indicators, PhA may serve as a more useful complementary evaluation tool in maintaining health and improving HRQoL in older adults.

## Introduction

The average life expectancy is steadily on the rise in developed countries, and the population is aging rapidly. Among these countries, Japan is the most rapidly aging country [1,2]. As of 2020, the number of people aged 65 years and older in Japan reached 35.9 million, accounting for 28.4% of the total population [2]. The proportion of people aged ≥65 years is estimated to continue to increase in the future [2]. Increasing attention is being paid to the decline in health-related quality of life (HRQoL) that accompanies aging, owing to factors such as the decline in immune function, hearing and vision, muscle strength, balance, and cognitive function [3]. It has been reported that low HRQoL is associated with an increased risk of chronic disease and mortality [4,5]. Therefore, it is important to identify and respond to the risk of declining HRQoL in older adults at an early stage [3,4].

The phase angle (PhA) has attracted attention in recent years as an indicator of the quality of skeletal muscle [6] and is measured using bioelectrical impedance analysis (BIA) [7]. PhA is associated with mortality in patients with heart failure [8] and is significantly associated with mortality in older adults [9]. As an indicator, it sensitively reflects nutritional status and is associated with a decline in nutritional status in older adults [10]. Many unknown factors are related to the QoL of community-dwelling older adults. These include walking speed, the five-times sit-to-stand test, balance, and grip strength [11,12]. However, there is no consensus on it so far, as it has also been reported that QoL is not related to balance or grip strength [11,13].

Regarding the relationship between PhA and QoL, as reported previously, the lower the PhA in a particular patient, the lower the QoL; moreover, the lower the PhA in patients with lumbar spinal stenosis [14], idiopathic pulmonary fibrosis [15], or cancer [16], the lower the QoL. Despite this link between PhA and QoL in specific patient populations, in community-dwelling older adults, the relationship between QoL and PhA remains unclear, and there is limited understanding of the PhA's role. Addressing this gap can help identify early interventions to prevent HRQoL decline. Therefore, this study aimed to examine the association between PhA, as measured by BIA, and HRQoL in community-dwelling older adults. We hypothesized that PhA is independently associated with HRQoL and may serve as a useful early indicator for identifying individuals at risk of HRQoL decline in this population.

## Materials and methods

Study design and participants 

This cross-sectional study included community-dwelling older adults aged 65-80 years. The evaluation of participants, recruited through local public notices, was conducted at a local venue. The selection criteria were as follows: independence in activities of daily living (ADL), and no serious musculoskeletal, neurological, or internal organ disorders that could interfere with the evaluation. Participants with cardiac pacemakers or those whose evaluations could not be performed using BIA were excluded. The evaluations were performed by physical therapists with extensive clinical experience in working with older adults. 

Per the Declaration of Helsinki, the purpose of the study, details of the examination, and protection of personal information were explained to the participants verbally and in writing, and written informed consent was obtained from all participants. This study was approved by the Kansai Medical University Medical Ethics Review Committee (approval number: 2023053). 

Evaluation methods

Questionnaire-Based Evaluation

HRQoL was assessed using the Japanese version of the EuroQol 5-Dimension 5-Level (EQ-5D-5L) questionnaire [17]. The EQ-5D-5L questionnaire consists of five questions on “mobility,” “personal care,” “usual activities,” “pain/discomfort,” and “anxiety/depression.” For each item, participants had to select the best option for describing their health condition from among five predefined options, ranging from no problems to severe problems. The final value for each selected health condition was calculated using a conversion table [18]. The distribution of the calculated values ranged between -0.025 and 1.000; the higher the value, the higher the HRQoL. 

Body Composition Assessment

**Figure 1:**
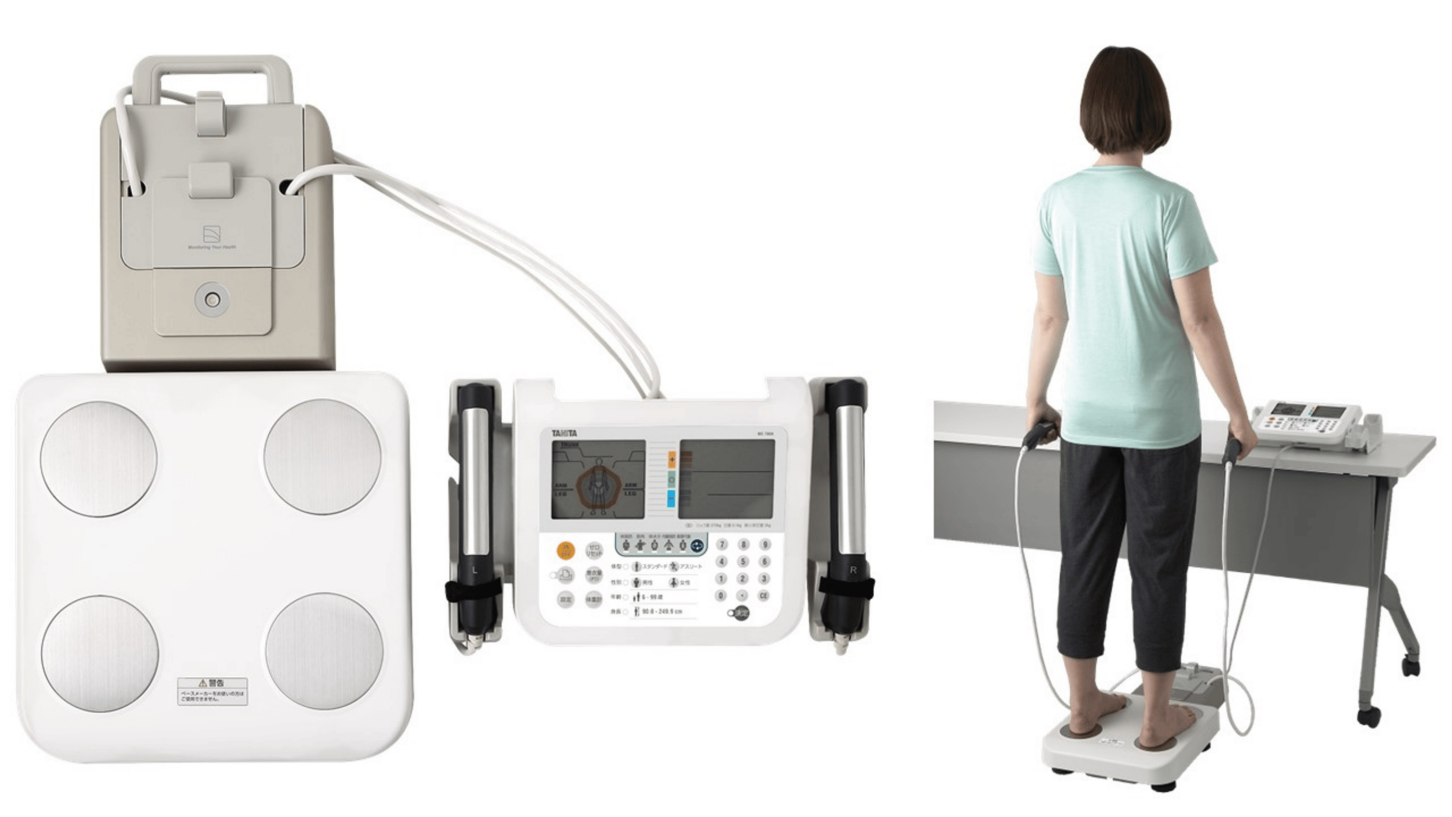
Body composition measurement The permission to publish Figure 1 was obtained from TANITA Corporation. The image rights belong to TANITA Corporation

A multifrequency BIA body composition analyzer (MC-780A-N; TANITA Corporation, Tokyo, Japan) was used to measure body composition (Figure 1). The BIA device measures electrical resistance by passing a weak alternating current (≤90 μA) through the body. The frequency of the current used was 50 kHz. PhA was calculated using the formula (−arc tangent(Xc/R) × (180/π)) (°) based on R and Xc at 50 kHz [19], and the average of the right and left halves of the body was used for the analysis. PhA is calculated directly from reactance and resistance, without estimation formulas or complex algorithms, making it a straightforward and relatively consistent measurement even between different devices. When measured according to standardized protocols, inter-rater variability is minimized, supporting its reliability as an assessment tool [20]. The participants stood barefoot on the measurement platform, held their handgrips, and maintained a standing posture for approximately 15 seconds while the measurements were taken. All BIA measurements were performed between 10:00 a.m. and 12:00 p.m., and participants were instructed to avoid eating or engaging in strenuous physical activity for at least two hours before the measurements to minimize the influence of hydration and metabolic factors on PhA values. The appendicular skeletal muscle mass index (ASM) was calculated by adding the masses of the upper and lower limbs on both sides. The skeletal muscle index (SMI) was calculated by dividing the ASM by the square of the height.

Evaluation of Physical Function

For physical function assessment, grip strength, normal walking speed, the five-times sit-to-stand test, and knee extension strength were evaluated. A Smedley dynamometer (T.K.K.5401; Takei Kiki Kogyo Co., Ltd., Niigata, Japan) was used to measure the grip strength. The measurement was performed in the standing position, and the participant was instructed to grip the handle with maximum force for approximately three seconds. The measurement was performed once using the dominant hand [21]. During the measurement, the participants were not allowed to hold their breath, and the dynamometer was held in a stable position. The maximum value displayed on the dynamometer was recorded.

For normal walking speed, the measurement was set at 10 m, with a 3 m reserve path at the front and back, resulting in a total walking path of 16 m. A stopwatch was used to measure the required time, which was converted to speed (m/s) [22]. No walking aids were used, and the participants were instructed to walk at their “usual speed.” All measurements were performed once. For the five-times sit-to-stand test, the participants were asked to sit on a chair 40 cm high with their legs spread out shoulder width apart and their arms folded in front of their chest. At the “start” signal, they were asked to stand up with their trunks and with both knee joints fully extended, and then return to the sitting position as quickly as possible. The time taken to complete five repetitions of the sit-to-stand test was measured, and only one measurement was recorded [23].

Knee extension strength was measured using a manual dynamometer (MT-201; Sakai Medical Co., Ltd., Tokyo, Japan). The sensor pad was attached to the front of the distal part of the lower leg using hook-and-loop fasteners while the participant was seated in a chair. The length of the belt was adjusted so that the lower leg was in a drooping position, and the lower leg was fixed to the back of the chair. The trunk was maintained in a vertical position, and the arms were folded in front of the chest. For participants with poor sitting balance, the examiner supported their trunk from behind. The examiner also held the sensor pad in place to prevent it from slipping during the measurement and verbally instructed the participants to extend their knees to the maximum extent for approximately three seconds. Measurements were performed twice on each leg, with at least 30 seconds between the measurements [24]. The average of the two measurements was used to indicate the knee extension strength divided by the body weight [24].

Other Measurement Items

Other measurement items included basic attributes (age and sex) obtained from the participants. BMI was calculated from height measured using a height meter and weight measured using a body composition analyzer.

Statistical analysis 

We performed the Shapiro-Wilk test [25] for each measurement to confirm the normality of the evaluation results. As all evaluation results were non-normally distributed, we examined the relationship between HRQoL and other measurement items using Spearman's rank correlation test [26]. Furthermore, multiple regression analysis was performed with HRQoL as the dependent variable [27]. To examine the factors affecting HRQoL, explanatory variables were incorporated into Models 1-3. The explanatory variables for Model 1 were PhA, sex, ASM, and grip strength. The explanatory variables for Model 2 were PhA, sex, grip strength, knee extension strength, and walking speed. The explanatory variables for Model 3 were PhA, sex, ASM, grip strength, knee extension strength, walking speed, and the five-times sit-to-stand test. Because ASM and PhA were lower in women than in men [6], we incorporated sex as a covariate in the analysis to control for the effect of sex. SPSS Statistics for Windows, version 29 (IBM Corp., Armonk, NY, USA) was used for statistical analysis, and the significance level was set at 5%. Given the exploratory design of this study, which aimed to examine potential associations in a community-dwelling older adult population, an a priori sample size calculation was not performed. The primary objective was to identify preliminary trends and generate hypotheses for future research, rather than test a specific effect size with predetermined statistical power.

## Results

Finally, data were collected for 162 subjects, and 159 subjects were included in the analysis, excluding three subjects with missing data. Basic characteristics of the participants and basic statistics for each measure are shown in Table 1. The mean age of the subjects was 77.8 years, 48 (30.2%) were male, and 111 (69.8%) were female. Other findings were as follows: ASM (16.3 ± 3.8 kg), HRQoL (0.88 ± 0.1), PhA (4.55 ± 0.6°), grip strength (23.0 ± 6.7 kg), walking speed (1.22 ± 2.0 seconds), five-times standing test (7.75 ± 2.0 seconds), and knee extension muscle strength (0.39 ± 0.19 kgf/kg).

**Table 1:** Participants’ basic characteristics and measurements ASM: appendicular skeletal muscle mass index; BMI: body mass index; HRQoL: health-related quality of life; PhA: phase angle; SMI: skeletal muscle mass index

Variables	Values
Categorical variables	N (%)
Male	47 (29.6%)
Female	112 (70.4%)
Continuous variables	Median [Q1 – Q3]
Age (years)	79 [74 – 82]
BMI (kg/m^2^)	22.1 [19.8 – 24.3]
PhA (°)	4.5 [4.0 – 4.9]
ASM (kg)	15.2 [12.8 – 17.4]
SMI (kg/m^2^)	6.45 [5.91 – 7.12]
HRQoL	0.89 [0.78 – 1.00]
Grip strength (kg)	20.6 [17.1 – 24.3]
Walking speed (m/s)	1.22 [1.07 – 1.38]
Five-time sit-to-stand test (s)	7.9 [6.3 – 9.5]
Knee extensor strength (kgf/kg)	0.35 [0.25 – 0.45]

The results of Spearman's rank correlation test for HRQoL and each measure are shown in Table 2. HRQoL was positively correlated with PhA (ρ=0.26) and weakly positively correlated with knee extension muscle strength (ρ=0.19), grip strength (ρ=0.16), and ASM (ρ=0.15).

**Table 2:** Spearman's rank correlation test results for HRQoL and each measure ^*^P<0.05. ^**^P<0.001 ASM: appendicular skeletal muscle mass index; BMI: body mass index; HRQoL: health-related quality of life; PhA: phase angle; SMI: skeletal muscle mass index

Parameter	ρ	P-value
Age	0.0279	0.7260
BMI	-0.0283	0.7230
PhA	0.2550	<0.0010^**^
ASM	0.1580	0.0443^*^
SMI	0.1200	0.1320
Grip strength	0.1610	0.0414^*^
Walking speed	0.0256	0.7480
Five-time sit-to-stand test	-0.0968	0.2250
Knee extensor strength	0.1670	0.0351^*^

The results of the multiple regression analysis with HRQoL as the objective variable are shown in Table 3. Model 1 found an association with PhA (β=0.0488, 95%CI [0.018, 0.078], t=3.19, p=0.001) but not ASM or grip strength; Model 2 found an association with PhA (β=0.0456, (β=0.0456, 95% CI [0.013, 0.078], t=2.76, p=0.006) and PhA (β=0.0452, 95% CI [0.012, 0.078], t=2.73, p=0.007) for Model 3, all showing an association between HRQoL and PhA, but not other physical functions. The results showed an association between HRQoL and PhA, but no association between HRQoL and other physical functions. In all models, PhA showed a statistically significant association with HRQoL, while no significant associations were observed with the other physical function indicators. However, the overall strength of the association was modest.

**Table 3:** Multiple regression analysis results of associated factors of HRQoL ^*^Significant association ASM: appendicular skeletal muscle mass index; HRQoL: health-related quality of life; PhA: phase angle

Model	Explanatory variable	Estimated value	95% confidence interval	t-value	P-value
Model 1	PhA	0.0487	0.0185 – 0.0790	3.1839	0.0017^*^
	Sex	-0.0473	-0.1133 – 0.0208	-1.3611	0.1754
	ASM	-0.0019	-0.0111 – 0.0067	-0.4878	0.6263
	Grip strength	0.0002	-0.0046 – 0.0051	0.1055	0.9160
Model 2	PhA	0.0451	0.0128 – 0.0774	2.7599	0.0064^*^
	Sex	-0.0415	-0.1021 – 0.0190	-1.3542	0.1776
	Grip strength	0.0001	-0.0041 – 0.0044	0.0716	0.9429
	Knee extensor strength	0.0449	-0.0626 – 0.1524	0.8250	0.4106
	Walking speed	-0.0321	-0.1243 – 0.0600	-0.6878	0.4925
Model 3	PhA	0.0463	0.0137 – 0.0788	2.8097	0.0056^*^
	Sex	-0.0476	-0.1154 – 0.0202	-1.3871	0.1674
	ASM	-0.0024	-0.0119 – 0.0071	-0.5014	0.6168
	Grip strength	0.0006	-0.0045 – 0.0056	0.2140	0.8308
	Knee extensor strength	0.0344	-0.0786 – 0.1475	0.6018	0.5482
	Walking speed	-0.0449	-0.1430 – 0.0531	-0.9058	0.3665
	Five-time sit-to-stand test	-0.0034	-0.0138 – 0.0069	-0.6504	0.5164

## Discussion

This study examined the relationship between HRQoL, PhA, and various physical functional indicators in community-dwelling older adults. The results showed that PhA was significantly associated with HRQoL, but not with ASM, grip strength, or knee extension strength, which are indicators of physical function. This suggests that PhA may be an important factor for evaluating HRQoL in older adults. This study is important because it analyzed the relationship between HRQoL and PhA and multiple physical function indicators from multiple perspectives in community-dwelling older adults. PhA is an indicator of skeletal muscle quality [6] and nutritional status [10], and is associated with mortality in patients with heart failure [8] and QoL in those with cancer [16]. In addition, PhA is associated with inflammatory markers, such as C-reactive protein and interleukin-6 [28], suggesting that it may be a useful tool for assessing nutritional and general health status in clinical practice. Studies on the relationship between QoL and PhA have mainly been conducted in specific patient populations, such as those with lumbar spinal stenosis [14], idiopathic pulmonary fibrosis [15], and cancer [16]. Very few studies have been conducted in healthy older adults.

Furthermore, previous studies of older adults living in the community have reported an association between QoL and walking speed, the five-times sit-to-stand test, and balance [11], as well as an association between QoL and grip strength [12]. However, there have also been reports of no association between QoL and balance [13], and studies reporting no relationship between QoL and grip strength [11]; therefore, there is currently a lack of consensus. This study provides new insights into previous findings by analyzing the relationship between HRQoL and PhA in community-dwelling older adults. PhA as an indicator reflects the quality of skeletal muscle and overall health status [6,28], and has the potential to evaluate HRQoL from a different perspective than the physical function indicators that have been reported to date.

In this study, PhA was significantly associated with HRQoL. The factors associated with PhA and HRQoL include cell membrane integrity, nutritional status, chronic inflammation, and skeletal muscle quality. By detailing these factors, it is possible to comprehensively understand the relationship between PhA and HRQoL. PhA is an indicator of cell membrane integrity and the distribution of water inside and outside the cell, and can be used to assess cellular health, including cell membrane integrity [29]. When PhA is high, the cell membrane is healthy, which optimizes tissue function and is thought to contribute to improved HRQoL [15]. However, when PhA is low, it suggests that the cell membrane is deteriorating, which may lead to a deterioration in overall health. Previous studies have shown that PhA is a sensitive indicator of nutritional status [10]. A low PhA is associated with malnutrition and sarcopenia [10], and maintaining an appropriate nutritional status in older adults may improve physical and mental performance and contribute to improved HRQoL.

Chronic inflammation is another important risk factor. Low PhA correlates with elevated inflammatory markers, such as C-reactive protein and interleukin-6, indicating chronic inflammation [28]. The higher the C-reactive protein and interleukin-6 levels, the lower the PhA, which may suggest that the higher the degree of chronic inflammation, the lower the integrity of the cell membrane. As chronic inflammation progresses, it can damage cells and tissues, which may reduce overall health and HRQoL [30]. Another important characteristic of PhA is that it can be used to assess skeletal muscle quality. Unlike conventional physical function indicators, such as ASM and grip strength, PhA is superior in that it not only evaluates muscle mass and muscle strength but also the quality of skeletal muscle [6].

PhA offers several practical advantages in clinical settings. For instance, it can be measured objectively using BIA, requires minimal subject cooperation, and can be completed in approximately 15 seconds [6]. Moreover, when using BIA, not only can it be measured quickly and non-invasively, but it is also less influenced by the subject’s effort, cognitive status, or emotional state [7]. In contrast, conventional assessments such as grip strength, walking speed, and HRQoL questionnaires like the EQ-5D depend on the subject’s understanding, motivation, or mood, and may be influenced by day-to-day variability or bias [4]. Therefore, BIA-derived PhA may serve as a useful tool for screening HRQoL decline risk, independent of these daily fluctuations. However, given that the association between PhA and HRQoL was modest in our study, we emphasize that PhA should be regarded as a complementary evaluation tool rather than a standalone indicator. The higher the PhA, the better the quality of skeletal muscle, and possibly, it shows a stronger association with HRQoL by reflecting health status at the cellular level.

HRQoL is a multidimensional construct influenced by physical, psychological, and social factors. PhA is an objective indicator that reflects cellular integrity, muscle mass, and nutritional status, and is associated with physical frailty and sarcopenia [10]. Physical impairments can lead to reduced independence and physical activity, which in turn may contribute to psychological deterioration such as depression and anxiety, as well as increased risk of social isolation [31]. Therefore, although PhA does not directly capture psychological or social dimensions, it may be indirectly related to them through its association with physical decline and may serve as a predictive marker for reductions in overall HRQoL.

In this study, no association was found between HRQoL and physical function indicators, such as ASM, grip strength, or knee extension strength. These results suggest that physical function indicators do not directly affect HRQoL, or that HRQoL may be affected by factors other than physical function. Previous studies have also reported that HRQoL is determined by a complex combination of psychosocial factors, living environment, and physical function [32]. ASM, grip strength, and knee extension strength are indicators that reflect individual aspects of physical ability, such as muscle mass and strength. These indicators do not directly measure ADL or the ability to perform social roles in older adults. Even if muscle strength declines in older adults, a high HRQoL can be maintained with appropriate social support and good psychological health. This could explain the lack of a direct relationship between the physical function indicators - ASM, grip strength, and knee extension strength - and HRQoL. However, this study did not examine the specific relationship between HRQoL and psychological or social factors, leaving these aspects unclear. To fully understand how psychosocial factors influence the relationship between HRQoL and physical function indicators, further research is needed to develop a comprehensive model that integrates these factors.

The major strength of this study is that it demonstrates the potential of PhA as a novel indicator for predicting the risk of decline in HRQoL. By focusing on PhA, which reflects cellular-level health status, this study provides a new perspective for a more comprehensive understanding of the health status of older adults. In particular, the fact that PhA was independently associated with HRQoL, independent of physical function indicators, provides practical information for health management and preventive measures for older adults. Another strength of this study is that by focusing on community-dwelling older adults, it provides important information that expands the scope of PhA application. The fact that this study also conducted a multifaceted analysis combining HRQoL and physical function indicators enhanced its authenticity. Associations between PhA and various health outcomes have been reported across a wide range of diseases and in multiple regions. This study provides important preliminary evidence supporting the potential clinical application of PhA in community-dwelling older adults. These findings suggest that the association between PhA and HRQoL may be applicable across different cultural and regional contexts; however, further research is warranted to validate its generalizability in more diverse populations.

One limitation of this study is that it utilized a cross-sectional design; therefore, it could not demonstrate causal relationships. Future research should employ a longitudinal or prospective cohort study design to examine the impact of PhA on HRQoL over time. Such a study design would enable a better understanding of temporal changes and identify potential causal relationships. Additionally, because this was an exploratory cross-sectional study, a priori sample size calculation was not conducted. As a result, the statistical power may have been insufficient to detect small or moderate associations, and the generalizability of the findings may be limited. Future studies should include appropriate sample size estimation to enhance the validity and applicability of the results. In addition, PhA measurements may be dependent on the BIA method. The BIA method is easily affected by factors such as measurement time. Therefore, a standardized protocol is required to improve PhA measurement accuracy. As the participants of this study were community-dwelling older adults in Japan, it is unclear whether similar results could be obtained in other cultural or regional settings. Therefore, comparative studies targeting older adults in other countries are needed.

## Conclusions

Our findings showed that when compared with other physical function and muscle strength indicators, PhA may serve as a more useful complementary evaluation tool in maintaining health and improving HRQoL in older adults. PhA may play an important role in early interventions and prevention measures to improve the HRQoL of community-dwelling older adults. Further validation of PhA through longitudinal and interventional studies is required to strengthen its role in clinical practice.
